# Genetic manipulation of SPG7 or NipSnap2 does not affect mitochondrial permeability transition

**DOI:** 10.1038/s41420-020-0239-6

**Published:** 2020-01-29

**Authors:** Paula J. Klutho, Ryan J. Dashek, Lihui Song, Christopher P. Baines

**Affiliations:** 1 Dalton Cardiovascular Research Center, University of Missouri, Columbia, MO 65211 USA; 2 Department of Veterinary Pathobiology, University of Missouri, Columbia, MO 65211 USA; 3 Department of Biomedical Sciences, University of Missouri, Columbia, MO 65211 USA; 4 Department of Medical Pharmacology and Physiology, University of Missouri, Columbia, MO 65211 USA

**Keywords:** Cell death, Mitochondrial proteins

Opening of the mitochondrial permeability transition (MPT) pore is known to mediate cellular necrosis in response to a number of toxic stimuli, such as elevated Ca^2+^ levels and oxidative stress, and therefore contributes to multiple pathologies^1^. However, the identity of the components that make up the channel-forming unit of the MPT pore remain uncertain, with many candidates being ruled out by genetic studies^2,3^, and only cyclophilin-D (CypD) confirmed as a key regulator of the MPT pore^2,3^. A previous study identified the mitochondrial AAA-protease subunit spastic paraplegia 7 (SPG7) as a novel modulator of the MPT pore^4^. They reported that SPG7 interacted with CypD and that depletion of SPG7 in HEK-293 cells greatly attenuated Ca^2+^ and oxidative stress-induced MPT and cell death. The authors concluded that SPG7 was an essential component of the MPT pore. They additionally reported that depletion of NipSnap2 (also known as Gbas), a mitochondrial protein of unknown function, also attenuated MPT. The latter was interesting, Halestrap’s group have also identified NipSnap2 as a CypD-binding protein^5^.

However, there are issues with the authors’ conclusions. As pointed out by others^6^, MPT still occurs in the SPG7 deficient cells albeit at higher Ca^2+^ concentrations. Consequently, the authors’ data would indicate that SPG7 is instead a positive regulator of the MPT pore, akin to CypD, rather than the essential channel-forming unit. Moreover, indirect effects cannot be ruled out and it has been suggested that SPG7’s ability to regulate MPT is through proteolysis of the Ca^2+^-import machinery, rather than a direct effect^7,8^. The discrepancies are further complicated by the report that depletion of SPG7 has no effect or even exacerbates MPT^7^. Thus, there is considerable controversy regarding a role for SPG7 in the regulation MPT. Moreover, there have been no studies aimed at reproducing the NipSnap2 experiments.

**Fig. 1:**
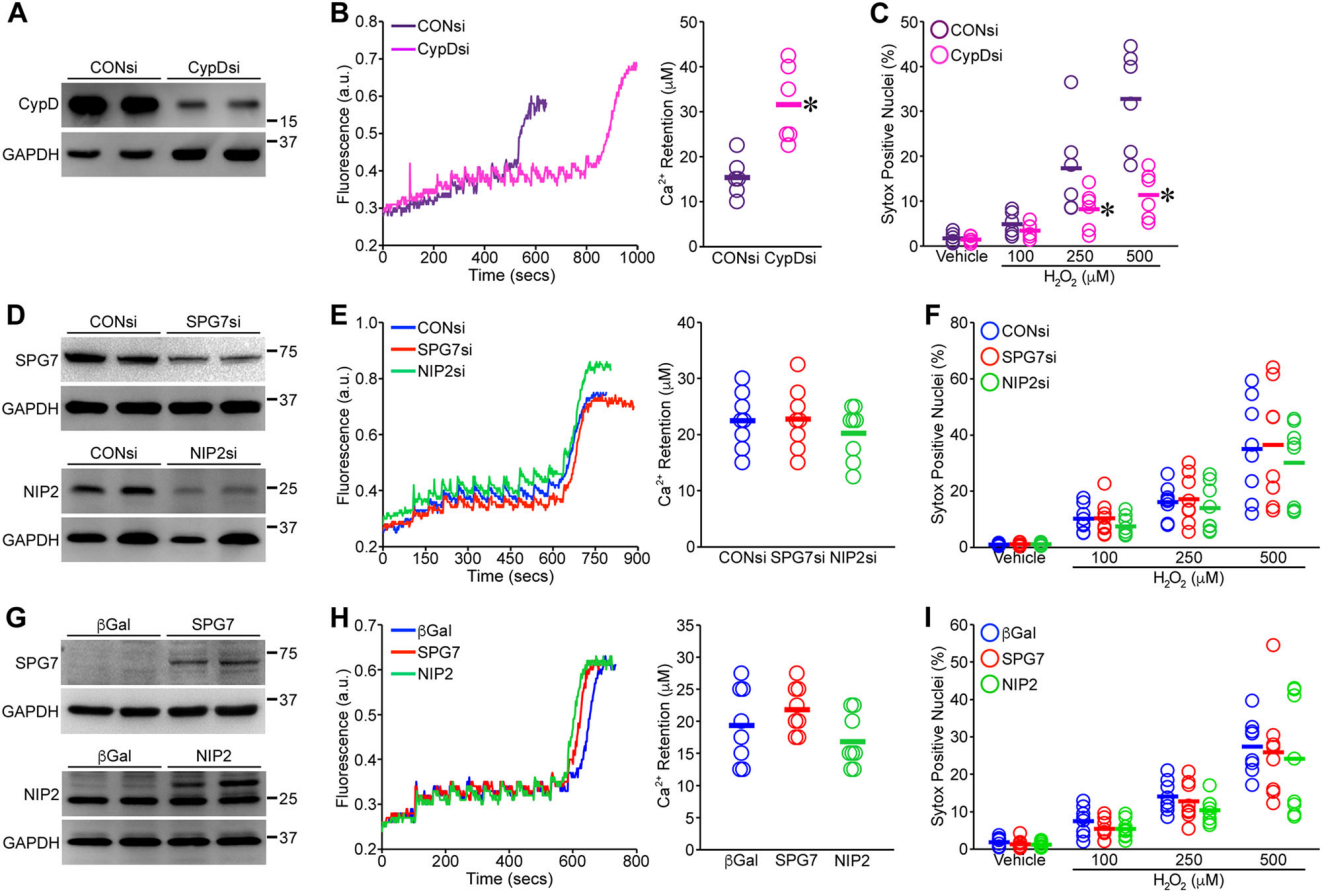
Manipulation of SPG7 or NipSnap2 expression does not alter MPT or oxidative stress-induced cell death. **a** Western blots for CypD and GAPDH in MEFs transfected with control or CypD siRNAs. **b** Representative traces and quantification of Ca^2+^ retention capacity (CRC) in permeabilized control and CypD siRNA-transfected MEFs. **c** Cell death as measured by Sytox staining in control and CypD siRNA-transfected MEFs exposed to increasing concentrations of H_2_O_2_ for 4 h. **d** Western blots for SPG7, NipSnap2, and GAPDH in MEFs transfected with control, SPG7, or NipSnap2 siRNAs. **e** Representative traces and quantification of CRC in permeabilized control, SPG7, and NipSnap2 siRNA-transfected MEFs. **f** Sytox staining in control, SPG7, and NipSnap2 siRNA-transfected MEFs exposed to increasing concentrations of H_2_O_2_ for 4 h. **g** Western blots for SPG7, NipSnap2, and GAPDH in MEFs infected with βGal, SPG7, or NipSnap2 adenoviruses. **h** Representative traces and quantification of CRC in permeabilized βGal, SPG7, and NipSnap2-infected MEFs. **i** Sytox staining in βGal, SPG7, and NipSnap2-infected MEFs exposed to increasing concentrations of H_2_O_2_ for 4 h. Each individual point represents one independent cell isolate. Bar represents the mean. Differences in CRC between two groups were assessed using an unpaired two-sided t-test or between three groups using One-Way ANOVA followed by Scheffé’s post-hoc test. Differences in cell death between groups were analyzed using Two-Way ANOVA followed by Scheffé’s post-hoc test. *p < 0.05 vs. CONsi.

To address this, we depleted (siRNA) and overexpressed (adenovirus) SPG7 or NipSnap2 in primary culture mouse-embryonic fibroblasts (MEFs) isolated from male and female C57BL/6J e15.5 embryos. MEF isolation was approved by the University of Missouri Animal Care and Usage Committee and was in accordance with the Guidelines for the Care and Use of Laboratory Animals published by the National Institutes of Health. We then assessed Ca^2+^-induced MPT and oxidative stress-induced death. First, as a positive control, we knocked down CypD (Fig. 1a) and measured MPT using the Ca^2+^-retention capacity (CRC) assay, confirming an attenuated MPT response in the CypD-depleted MEFs (Fig. 1b). Oxidative stress-induced necrosis is mediated in part by opening of the MPT pore and we demonstrated that CypD knockdown could markedly attenuate the degree of cell death to increasing concentrations of H_2_O_2_ (Fig. 1c).

In contrast to CypD, knockdown of SPG7 or NipSnap2 (Fig. 1d) had no observable effect on the MPT response to Ca^2+^ in MEFs (Fig. 1e). Consistent with this finding, depletion of either protein did not alter the cell death response to H_2_O_2_ (Fig. 1f). We then tested if overexpression of SPG7 or NipSnap2 altered MPT and cell death. Increased levels of SPG7 or NipSnap2 (Fig. 1g) did not affect CRC in the permeabilized fibroblasts (Fig. 1h). Overexpression of the two proteins similarly failed to affect H_2_O_2_-induced cell death (Fig. 1i).

Thus, unlike the report by Shanmughapriya et al.^7^, we could not demonstrate a role for either SPG7 or NipSnap2 in the regulation of the MPT pore. This would be more in line with the study by König and colleagues. One difference is that the former study primarily utilized 293 and HeLa cells, which are immortalized and, thus, are essentially a single biological replicate. That being said, the studies by König et al.^7^ and Hurst et al.^8^ also utilized HEK-293s for their knockdown/knockout approaches. In contrast, we used separate primary MEF isolates and thus have 7–8 biological replicates. In addition, we utilized an acute knockdown approach as opposed to the long-term reductions in SPG7 or NipSnap2 (shRNA and CRISPR) in the other studies^4,8^. Hence compensatory changes due to chronic loss of SPG7 cannot be ruled out. For example, stable SPG7 shRNA knockdown or SPG7 knockout cells exhibited increased ATP levels^4^, which is known to reduce MPT pore opening^9^. However, we observed the opposite with a modest reduction in ATP with acute SPG7 depletion (0.85 ± 0.07 fold, *p* = 0.010) and an increase in ATP with overexpression (1.78 ± 0.29 fold, *p* = 0.033). Knockdown or overexpression of NipSnap2 did not alter cellular ATP levels. Shanmughapriya et al. did perform acute SPG7 knockdown experiments in cardiac myocytes and reported inhibition of MPT^4^. It could be that there are cell type differences, although this would also argue against SPG7 being essential as presumably the pore-forming unit is conserved between cell types.

We cannot rule out that off-target effects of the siRNAs could potentially contribute to the different phenotypes^10^, although this is mitigated by the fact that we used pools of four independent siRNAs for each target^10^. It is also feasible that redundancy between isoforms may be at play. For example, NipSnap2 is highly homologous to NipSnap1^11^. SPG7 is a component of the mitochondrial mAAA-protease, forming hetero-oligomers with AFG3L2^12^. However, the protease can still function in the absence of SPG7 due to homo-oligomerization of AFG3L2^12^. Interestingly, Shanmughapriya et al. also pulled out AFG3L2 as potential MPT mediator in their screen, although the effect of its depletion was not as profound as that with SPG7^4^. However, in the study by König et al. simultaneous depletion of AFG3L2 and SPG7 significantly exacerbated the MPT response^7^. The future use of an inducible CRISPR-Cas9 system may help circumvent these issues as well as bypass any compensatory effects of chronic knockdown/knockout of the proteins.

In conclusion, based upon our data neither SPG7 nor NipSnap2 appear to be essential components or even regulators of the MPT pore and efforts to identify the channel-forming unit need to be re-directed.
